# Chlorination Disinfection By-products and Pancreatic Cancer Risk

**DOI:** 10.1289/ehp.7403

**Published:** 2005-01-10

**Authors:** Minh T. Do, Nicholas J. Birkett, Kenneth C. Johnson, Daniel Krewski, Paul Villeneuve, the Canadian Cancer Registries Epidemiology Research Group

**Affiliations:** ^1^Department of Public Health Sciences, University of Toronto, Toronto, Ontario, Canada; ^2^Department of Epidemiology and Community Medicine, University of Ottawa, Ottawa, Ontario, Canada; ^3^McLaughlin Centre for Population Health Risk Assessment, Institute of Population Health, University of Ottawa, Ottawa, Ontario, Canada; ^4^Population and Public Health Branch, Health Canada, Ottawa, Ontario, Canada; ^5^Epistream Consulting Inc., Ottawa, Ontario, Canada

**Keywords:** chlorination by-products, drinking water, pancreatic cancer, THMs

## Abstract

Chlorination disinfection by-products (CDBPs) are produced during the treatment of water with chlorine to remove bacterial contamination. CDBPs have been associated with an increased risk of bladder cancer. There is also some evidence that they may increase the risk of pancreatic cancer. We report results from a population-based case–control study of 486 incident cases of pancreatic cancer and 3,596 age- and sex-matched controls. Exposure to chlorination by-products was estimated by linking lifetime residential histories to two different databases containing information on CDBP levels in municipal water supplies. Logistic regression analysis found no evidence of increased pancreatic cancer risk at higher CDBP concentrations (all odds ratios < 1.3). Null findings were also obtained assuming a latency period for pancreatic cancer induction of 3, 8, or 13 years.

Chlorine has been used as a disinfection agent for raw water supplies since the early 1900s (Wigle 1998). Chlorination is presently the most common procedure used for water treatment worldwide. Its widespread use has been credited with largely eliminating the risk of illnesses caused by cholera and other microbiologic contaminants in drinking water. Despite the effectiveness of chlorine in preventing morbidity and mortality due to water-borne pathogens, there remains concern about possible adverse health effects associated with chronic exposure to chlorination disinfection by-products (CDBPs) present in drinking water and, in particular, about the carcinogenic potential of CDBPs (Bellar et al. 1974; Krewski et al. 2002; Rook 1974).

Chlorine reacts with naturally occurring organic material in raw water supplies to produce a variety of CDBPs that can be grouped together based on molecular structure. The most common are trihalomethanes (THMs), haloacetic acids, and haloacetonitriles. THMs (the most abundant CDBP) consist of four species: chloroform (TCM), bromodichloromethane (BDCM), dibromochloromethane, and bromoform.

Although animal studies have consistently shown an association between THMs and both liver and kidney cancer (Dunnick and Melnick 1993), evidence of human carcinogenicity is limited. In a 1992 review of the literature concerning cancer risk after CDBP exposure, Morris et al. (1992) reported evidence of an increased risk only for bladder cancer [odds ratio (OR) = 1.41; 95% confidence interval (95% CI), 1.25–1.62] and rectal cancer (OR = 2.04; 95% CI, 1.16–3.53). Subsequently, three population-based case–control studies of CDBPs and bladder cancer reported statistically elevated risk with ORs in the range of 1.6–1.8 (Cantor et al. 1998; King and Marrett 1996; McGeehin et al. 1993). However, the increased risk for colon cancer identified by Morris et al. (1992) has not been confirmed in later studies (King et al. 2000).

The role of CDBPs in the etiology of pancreatic cancer has been less well investigated. Pancreatic cancer is the fourth leading cause of cancer mortality in Canada and the United States, with an annual incidence rate of approximately 9/100,000 (Ahlgren 1996; National Cancer Institute of Canada 2002). The etiology of pancreatic cancer remains largely unknown, with only age and tobacco smoking having been consistently identified as risk factors for this lesion (Ghadirian et al. 2003; Risch 2003). Chronic pancreatitis, obesity, diabetes mellitus, excess alcohol intake, meat intake, and reproductive factors in women have also been linked to pancreatic cancer risk, although the epidemiologic evidence is somewhat inconsistent (Anderson et al. 2002; Kreiger et al. 2001; Risch 2003).

In their review, Morris et al. (1992) identified six studies of CDBPs and pancreatic cancer. A combined analysis from these studies yielded a pooled OR of 1.05 (95% CI, 0.91–1.22). In a case–control study involving 101 cases, IJsselmuiden et al. (1992) subsequently found an OR of 2.23 (95% CI, 1.20–3.95) for people using municipal (chlorinated) water compared with people using nonmunicipal (nonchlorinated) water. In contrast, a case–control study conducted by Kukkula and Lofroth (1997) found a reduced risk of pancreatic cancer (OR = 0.20; 95% CI, 0.04–0.94) among those with chlorinated municipal drinking water from a surface source.

In this article, we report the results of a large population-based case–control study of incident pancreatic cancer cases derived from the National Enhanced Cancer Surveillance System (NECSS) of Canada. Individual exposures were estimated using two different databases containing information on CDBPs in municipal drinking water in Canada. One database provided time-dependent total THM concentrations, whereas the other provided single estimates of exposure to two specific THMs (TCM and BDCM) representing the entire study period.

## Materials and Methods

### Recruitment of subjects.

The NECSS was a collaborative effort between Health Canada and the Provincial Cancer Registries, which recruited, between 1994 and 1997, a total of 20,755 incident cases of 18 types of cancer (including 628 cases of pancreatic cancer) and 5,039 population-based controls who were frequency matched to the overall case group on age (5-year groups) and sex. Permission to contact the subjects was obtained from the attending physician. Two different strategies were used to identify controls: random selection from universal provincial health insurance plan rosters or provincial tax assessment roles, and random digit dialing. Interviews were conducted by mail, with the study questionnaire being mailed to cases within 1–4 months of diagnosis. Three of the eight participating provinces (Ontario, Nova Scotia, and Alberta) allowed the questionnaire to be completed by a proxy, most often a spouse, when the case had died or was too ill to complete the questionnaire. Overall, 24.8% of interviews for cases were provided by proxies. Because excluding proxy responses from the analysis did not affect the results, we retained proxy responses in all analyses reported in this article. Further details of the NECSS study are provided by Johnson and colleagues (Johnson 2000; Johnson et al. 1998).

The primary analytic sample for the present study was restricted to subjects between 30 and 75 years of age who lived in one of six Canadian provinces (Nova Scotia, Ontario, Manitoba, Saskatchewan, Alberta, and British Columbia) at the time of interview. Prince Edward Island and Newfoundland were excluded from the primary analyses because of small numbers. Of the cases who were mailed a questionnaire, 70% agreed to participate. The response rate among contacted controls was 65%. Cases were eligible for the study if this was their first cancer and their cancer diagnosis codes were either 157 [*International Classification of Diseases*, 9th Revision; World Health Organization (1977)] or C25 [*International Classification of Oncology*, version 2; Percy et al. (1990)]. Subjects were excluded if they had missing or invalid age information (*n* = 9), missing province of residence (*n* = 4), or inconsistent residence information (*n* = 292). One case who did not have pancreatic cancer was also excluded. This yielded a study sample size, before excluding subjects with missing values, of 576 cases and 4,105 controls.

The survey instrument contained questions about lifestyle factors, anthropometric measures, a 69-item food frequency questionnaire, and a detailed residence history. This last component asked subjects to record every address where they had lived at for at least 12 months, from birth to the date of the interview. For each location at which the subjects had lived, they were asked to record the time period (year they moved into the residence and the year they left the residence), street, city, county/ district, province, postal code (if available), and the main source of drinking water (town, dug well, drilled well, bottled, other).

### Exposure measurement.

CDBP exposure was estimated by linking information about residences to databases providing estimates of CDBP levels in municipal water supplies. All people receiving water from the same source were assumed to have been exposed to the same CDBP levels. Two linkage files were used. The first, the Environmental Health Directorate (EHD) file, provided summary information about three CDBPs (THM, TCM, and BDCM) but did not provide residence-specific exposures, which limited analysis of latency periods. The second, the Provincial and Municipal Water Monitoring (PMW) file, contained information about only one CDBP (THM) but included data on THM levels for different residences occupied by the study subjects. The EHD file was created by Health Canada using estimates of CDBP levels based on four surveys of water treatment facilities in 650 municipalities that had been conducted in 1962, 1975, 1988, and 1995. These surveys were supplemented by estimates from the 1993 National Survey of Chlorination Disinfection By-products, which collected water samples from 53 major cities in Canada and measured CDBP levels in these samples (Health Canada 1995). Health Canada had access to residence-specific information that was used to estimate average CDBP exposure levels between 1940 and 3 years before the interview. Although this average exposure metric was available to us for analysis, the residence-specific levels were unavailable for reasons of confidentiality.

The second linkage file was based on public domain information (the PMW file) (King and Marrett 1996). This file was based on reports from municipal water treatment facilities between 1990 and 1993. In order to extend the geographic coverage of this file, we used the Municipal Water Use Database (MUD) to estimate THM concentrations in drinking water (King et al. 2000). The MUD does not directly contain information about CDBPs. Rather, it contains information about water source and treatment practices for all communities in Canada with a population of at least 1,000. A linear regression model with an *R*^2^ of 0.76 was used to predict THM levels in a community, based on water source, treatment practices, and hydrologic characteristics of the community (King W, personal communication). Although only total THM levels could be estimated from the PMW file, information was available for each residence occupied by the study subjects.

Because both files provide an estimate of THM exposure in the 30-year exposure time window (ETW) ending 3 years before interview, we compared the estimates to document the similarity of the estimation methods. The Pearson correlation between the THM concentrations was 0.96. The concordance in classification of THM concentrations into the exposure groups used in our analyses was also high, with Cohen’s (unweighted) kappa being 0.82.

Individual exposure assignment was based on a predetermined ETW of 30 years ending 3 years before the year of interview. In most instances, the ETW corresponds to the period 1963–1993. Subjects using well water or bottled water as their primary source of water were assigned a THM exposure of zero for that residence. There was no information provided about the use of charcoal filters that might reduce CDBP levels. For the EHD file, the CDBP concentrations available to us represented a 40-year exposure period. This was adjusted to a 30-year ETW by computing a weighted average of the average THM exposure for years lived in a residence using municipal water and a value of zero for years lived in residence with well or bottled water. For the PMW file, exposure was obtained by averaging the annual exposure levels for each of the years within the ETW.

CDBP levels were analyzed after categorizing the exposure into four groups based on cut-points used in previously published studies. For THM, the exposure groups were < 10 μg/L, 10–20 μg/L, 20–50 μg/L, and > 50 μg/L. For TCM, the exposure groups were < 3 μg/L, 3–10 μg/L, 10–30 μg/L, and > 30 μg/L. For BDCM, the exposure groups were < 1 μg/L, 1–3 μg/L, 3–5 μg/L, and > 5 μg/L. The referent group was the lowest exposure group in all analyses.

In a secondary analysis we examined THM levels in the PMW file weighted by reported weekly intake of tap water. In this analysis, THM exposure was divided into quartiles, with the upper quartile being further divided into two groups using the 50th percentile of exposure within the upper quartile. (We do not provide the numerical cut-points because the weighted THM levels have no direct interpretation.)

Dietary factors (total daily caloric intake and total daily fat intake) were estimated using the method used by Villeneuve et al. (1999).

### Analysis.

The distributions of the exposures and potential confounders were explored using descriptive statistics. Unconditional logistic regression analysis was used to explore the effect of CDBP exposure on pancreatic cancer risk. All models adjusted for the three matching variables (age group, sex, and province of residence) and for body mass index (BMI); percent weight change; smoking, coffee, beer, liquor, and total fat intake; and energy intake. Analyses restricted to females were also adjusted for age at first menstruation and number of pregnancies. All continuous confounders were categorized into quartiles before analysis based on their distribution in the combined group of cases and controls. Effect modification by sex was explored by fitting separate logistic regression models for males and for females.

Observed control mean imputation (OCMI) (Weinberg et al. 1996) was used to impute exposures when subjects had missing or incomplete residence information. In this method, subjects for whom a CDBP exposure could not be computed because of missing data were assigned a CDBP level based on the average CDBP concentration observed in the control group. For the EHD file, imputation was done only when subjects failed to report their residence for ≥1 year. For the PMW file, exposures were imputed if a residence had been omitted or if there was no THM level available in the THM reference file for a specific residence. As a result, the level of imputation was higher in the PMW file (35–50%) than in the EHD file (24%). The effect of imputation was examined by comparing the OCMI-based results with those obtained using case-wise deletion of subjects with missing THM exposures. The two analyses produced similar results: although some ORs changed by 15–20%, overall statistical significance and exposure–response trends did not change appreciably. Consequently, we report here only those results based on OCMI.

The effect of exposure latency was explored using the annual exposure data available in the PMW file. Three analyses were conducted using latency periods of 3, 8, and 13 years. For example, in the 8-year latency analysis, exposure was based only on residences occupied in the period 8–33 years before interview. The EHD analyses incorporated a fixed 3-year latency period in the exposure assessment.

## Results

The study population included 576 cases and 4,105 controls, with a male-to-female case ratio of 1.29 (Table 1). As expected, the number of pancreatic cancer cases increased with age. The results of univariate logistic regression modeling of selected covariates (adjusted for age, sex, and province) are shown in Table 1. These results confirm the increased risk of pancreatic cancer associated with smoking, with the risk being significantly elevated in the highest two quartiles of smoking. There was some evidence of increased risk of pancreatic cancer in relation to higher body mass index (BMI; for BMI > 30 kg/m^2^, OR = 1.45; 95% CI, 1.11–1.91). There was a consistently lower risk of pancreatic cancer in relation to larger discrepancies between peak lifetime weight and weight reported 2 years before interview (OR = 0.72; 95% CI, 0.57–0.90). Beer (OR = 1.41; 95% CI, 1.09–1.84) and liquor intake (OR = 1.34; 95% CI, 1.03–1.75) were associated with increased pancreatic cancer risk, although the latter effect appeared to be confined to males. Wine consumption was not associated with pancreatic cancer risk in either men or women. There was some evidence of an increased risk of pancreatic cancer in relationship to total fat and total caloric intake, although these results were of marginal statistical significance. Consumption of tap water was not associated with pancreatic cancer. Neither educational attainment nor age at menarche was related to pancreatic cancer risk. Women who reported more than four pregnancies were at reduced risk of pancreatic cancer; this result is explored more fully by Kreiger et al. (2001).

Multivariate models (data not shown) confirmed the increased risk associated with smoking and the decreased risk associated with weight discrepancy. The effect of alcohol was largely eliminated after multivariate adjustment for other covariates.

Average THM levels in drinking water consumed by the study subjects varied by province, ranging from a low of 17 μg/L in Ontario to a high of 74 μg/L in Manitoba. Subjects in Manitoba also had the highest average levels of TCM (66 μg/L compared with between 11 and 22 μg/L in other provinces). BDCM exposure was more homogeneous, ranging from about 1.5 μg/L in British Columbia and Alberta to 5.8 μg/L in Manitoba and Saskatchewan. Average total THM levels for males and females were similar (25.2 vs. 23.7 μg/L, respectively), as were TCM (19.9 vs. 18.6 μg/L) and BDCM levels (3.2 vs. 3.2 μg/L). CDBP levels were similar across the study age groups, ranging from 23 to 25 μg/L for THM, from 18.4 to 20.4 μg/L for TCM, and around 3.1 μg/L in all age groups for BDCM. The mean CDBP levels were similar in the cases and controls: 24.3 versus 24.5 μg/L for THM, 19.5 versus 19.3 μg/L for TCM, and 3.1 versus 3.2 μg/L for BDCM.

ORs for pancreatic cancer risk in relation to exposure to THMs, BDCMs, and TCMs based on the EHD data file are shown in Table 2. Overall, these results provide little evidence of an association between CDBPs and pancreatic cancer. None of the tests for heterogeneity among the ORs in the CDBP exposure categories was statistically significant, nor was there any evidence of increasing trend in the ORs with increasing CDBP levels. For two analyses (THM in females and TCM for males), the test for heterogeneity approached a nominal *p*-value of 0.05. However, none of the ORs was significantly elevated, and there was no exposure gradient.

ORs for pancreatic cancer risk in relationship to THM levels based on the PMW data file are shown in Table 3. As with the EHD file, there was no evidence of heterogeneity in risk among the THM exposure categories for any of the three latency periods considered. The ORs did not suggest a pattern of increasing risk with higher THM levels.

The effect of weighting the level of THM exposure by a self-reported estimate of the amount of tap water drunk per day is shown in Table 4. All of the ORs were close to 1.0, with none of the results being statistically significant.

## Discussion

In the present study we found no evidence of an association between exposure to CDBPs in drinking water and the risk of pancreatic cancer. We did detect a significant effect of smoking on pancreatic cancer risk, although the magnitude of the risk was lower than has been reported in other studies. We also observed a reduction in risk in people who reported that their weight 2 years before their interview was lower than their peak lifetime weight.

The present analysis, based on about 480 incident pancreatic cancer cases, represents the largest such study conducted to date, notably larger than the previous studies of IJsselmuiden et al. (1992) and Kukkula and Lofroth (1997), which involved 101 and 183 incident cases, respectively. Although some studies based on decedent cases have had substantially larger case groups (up to 4,500), there is risk of bias when using the residence at the time of death as the basis for estimating lifetime CDBP exposure. Our study, which was based on estimates of average annual exposure based on self-reported lifetime residence histories, should provide a better indication of exposure to CDBPs in drinking water. Given the strength of our exposure data and the large sample size, our results suggest that CDBP exposure is unlikely to be a major risk factor for pancreatic cancer. The mean level in our study of the most common CDBP (THM, 24 μg/L) is similar to that reported in other studies from Iowa (Cantor et al. 1998) and Colorado (McGeehin et al. 1993)

Since 1978, 11 studies have explored the relationship of CDBPs and pancreatic cancer. These include two ecologic studies (Flaten 1992; Koivusalo et al. 1995), two cohort studies (Koivusalo et al. 1997; Wilkins and Comstock 2004), and seven case–control studies, of which five relied on decedent cases (Alavanja et al. 1978; Brenniman et al. 1978; Gottlieb et al. 1982; Young et al. 1981; Zierler et al. 1986) and the other two used incident cases (IJsselmuiden et al. 1992; Kukkula and Lofroth 1997). Exposure estimates in all of these case–control studies were based on CDBP levels at a single residence, in many cases relying on a coarse classification of whether or not municipal water was available in the house.

The two case–control studies that used incident cases found conflicting results. IJsselmuiden et al. (1992) found an elevated risk associated with CDBP exposure (OR = 2.18; 95% CI, 1.20–3.95). Although Kukkula and Lofroth (1997) reported a protective effect in their case–control study, this has not been replicated in other studies of pancreatic cancer. Neither of the cohort studies employed individual exposure measures; rather, exposure was inferred from access to municipal water supplies.

Wilkins and Comstock (2004) followed a cohort of 31,000 subjects for 12 years, with exposure status determined at recruitment. No significant differences were observed between subjects on municipal or nonmunicipal water sources (relative risk = 0.80; 95% CI, 0.44–1.52). Koivusalo et al. (1997) followed a cohort of 621,431 Finish residents for 23 years and found no evidence of an increased risk of pancreatic cancer (OR = 1.01; 95% CI, 0.70–1.20), with CDBP exposure inferred on the basis of the mutagenicity of communal water supplies.

Most previous studies estimated CDBP exposure on the basis of information from a single residence, under the assumption that this accurately reflects exposures from previous residences. In contrast, our study is based on annual CDBP exposures derived from lifetime residence histories. By linking each residence to local water supply information, we were able to account for regional variation in chlorination practices and water sources. For one of our two exposure classifications (the EHD file), we were able to estimate exposure based on measured CDBP levels in drinking water at the time the person was living at the residence. We compared the estimated mean THM exposure using a 30-year ETW to that based on exposure in the residence occupied 3 years before interview. The two estimates demonstrated a Pearson correlation coefficient of *r* = 0.96, and the ORs obtained using these two exposure estimates were generally similar and nonsignificant. Exposure assessment based on lifetime residence histories is preferred because temporal variation in CDBP concentrations is taken into account. Long-term average exposure levels can provide a good indicator of lifetime risk, particularly if variation in exposure levels over time is moderate (Goddard et al. 1995). The high correlation between results based on our two exposure databases suggests that previous studies based on a single estimate taken at a time point before diagnosis can be informative. The NECSS included a single estimate of the amount of tap water drunk by each subject. Although this did not permit a complete estimation of water consumption at the individual level because water contained in preparation of food and beverages was not considered, it did permit an analysis in which exposures were weighted by the amount of tap water consumed. This weighted analysis did not yield statistically significant increases in pancreatic cancer risk in relation to CDBP intake (Table 4). Nor did our study find an increased risk among people who reported drinking higher numbers of glasses of tap water per day (Table 1). Although it is possible that better measures of total water intake could yield more refined exposure estimates that would reveal some misclassification of total lifetime CDBP exposure in our study, the lack of suggestive evidence for any impact on risk of adjustment for tap water intake makes it unlikely that our analysis is missing important risk effects.

In addition to ingestion, people can be exposed to CDBPs from two other routes: inhalation and dermal contact. Although we had no information on which to estimate exposure from these alternate routes, evidence suggests that these routes of exposure could be important sources of CDBP exposure. In particular, Backer et al. (2000) found that a 10-min shower led to higher blood levels of CDBPs than did drinking 1 L of water. Hence, it is likely that our study, like all previous studies that have considered CDBP exposure only by ingestion, has underestimated total CDBP exposure. However, the analyses we performed were based on the rank ordering of intake, not on absolute intake levels. If this rank ordering is unaltered by the inclusion of dermal and inhalation exposure to CDBP, our risk estimates would remain valid. Nonetheless, consideration of CDBP exposures from multiple routes would be useful in future studies.

Although total THM levels are widely accepted as a marker of total CDBP concentrations, drinking water contains > 100 CDBPs. The present study is based on information on only three (THM, TCM, and BDCM) of the many known CDBPs. If other CDBPs demonstrate carcinogenic potency greater than that of THMs, TCMs, or BDCMs, it is possible that drinking water may be associated with cancer risk. However, because no association was found between intake of tap water and pancreatic cancer risk, the present study does not suggest the presence of other more potent CDBPs in drinking water. This hypothesis is supported by the lack of an association between the mutagenicity of drinking water and cancer risk (Koivusalo et al. 1997). Nonetheless, the possibility that there may exist a potent carcinogenic CDBP present at low levels in drinking water cannot be ruled out.

## Figures and Tables

**Table 1:** Descriptive statistics and ORs for selected subject characteristics.

	Male and female			Male			Female		
Characteristic	Cases*^a^*	Controls*^a^*	OR*^b^* (95% CI)	Cases*^a^*	Controls*^a^*	OR*^c^* (95% CI)	Cases*^a^*	Controls*^a^*	OR*^c^* (95% CI)
Sex									
Male	324 (56)	2,066 (50)	Matching variable						
Female	252 (44)	2,039 (50)							
Both	576	4,105							
Age (years)									
< 50	67 (12)	1,062 (26)		36 (11)	417 (20)		31 (12)	645 (32)	
50–54	52 (9)	358 (9)		29 (9)	132 (6)		23 (9)	226 (11)	
55–59	77 (13)	417 (10)	Matching variable	45 (14)	192 (9)	Matching variable	32 (13)	225 (11)	Matching variable
60–64	107 (19)	614 (15)		62 (19)	317 (15)		45 (18)	297 (15)	
65–69	138 (24)	804 (20)		77 (24)	481 (23)		61 (24)	323 (16)	
≥70	135 (23)	850 (21)		75 (23)	527 (26)		60 (24)	323 (16)	
All	576	4,105		324	2,066		252	2,039	
Smoking (pack-years)									
0	180 (32)	1,548 (38)	1.00*^d^*	70 (23)	525 (26)	1.00*^d^*	110 (45)	1,023 (51)	1.00*^d^*
1–15	115 (21)	1,197 (30)	0.86 (0.67–1.11)	61 (20)	630 (31)	0.70 (0.49–1.01)	54 (22)	567 (28)	1.04 (0.73–1.47)
15–35	167 (30)	872 (22)	1.55 (1.22–1.96)	107 (34)	538 (27)	1.35 (0.97–1.89)	60 (24)	334 (17)	1.79 (1.26–2.52)
> 35	95 (17)	411 (10)	1.76 (1.32–2.35)	73 (23)	330 (16)	1.66 (1.15–2.41)	22 (9)	81 (4)	2.07 (1.23–3.49)
Missing	19	77		13	43		6	34	
All	576	4,105		324	2,066		252	2,039	
BMI (kg/m^2^)									
< 23	146 (26)	1,150 (28)	1.00*^d^*	59 (18)	396 (19)	1.00*^d^*	87 (35)	754 (37)	1.00*^d^*
23–27	203 (36)	1,618 (40)	0.87 (0.69–1.09)	127 (40)	915 (45)	0.89 (0.64–1.24)	76 (30)	703 (35)	0.79 (0.57–1.10)
27–30	104 (18)	749 (18)	0.93 (0.71–1.23)	71 (22)	444 (22)	0.99 (0.68–1.44)	33 (13)	305 (15)	0.76 (0.50–1.17)
> 30	118 (21)	566 (14)	1.45 (1.11–1.91)	63 (19)	299 (15)	1.30 (0.88–1.93)	55 (22)	267 (13)	1.63 (1.12–2.37)
Missing	5	22		4	12		1	10	
All	576	4,105		324	2,066		252	2,039	
Percent weight change*^e^*									
< 2.9	187 (33)	930 (23)	1.00*^d^*	108 (34)	529 (26)	1.00*^d^*	79 (32)	401 (20)	1.00*^d^*
2.9–5.7	108 (19)	1,014 (25)	0.53 (0.41–0.68)	65 (20)	571 (28)	0.57 (0.41–0.79)	43 (17)	443 (22)	0.49 (0.33–0.73)
5.7–10.2	140 (25)	1,147 (28)	0.64 (0.50–0.81)	72 (23)	530 (26)	0.66 (0.48–0.92)	68 (27)	617 (31)	0.59 (0.41–0.84)
> 10.2	133 (23)	971 (24)	0.74 (0.58–0.95)	74 (23)	411 (20)	0.92 (0.66–1.27)	59 (24)	560 (28)	0.59 (0.41–0.86)
Missing	8	43		5	25		3	18	
All	576	4,105		324	2,066		252	2,039	
Coffee (cups)									
≤4 cups/week	64 (11)	513 (13)	1.00*^d^*	30 (9)	217 (11)	1.00*^d^*	34 (14)	296 (15)	1.00*^d^*
5–7 cups/week	146 (26)	1,131 (28)	0.87 (0.63–1.19)	71 (22)	575 (28)	0.77 (0.48–1.23)	75 (30)	556 (28)	0.98 (0.64–1.53)
2–3 cups/day	204 (36)	1,538 (38)	0.87 (0.64–1.18)	122 (38)	775 (38)	0.93 (0.60–1.44)	82 (33)	763 (38)	0.78 (0.51–1.21)
> 3 cups/day	152 (27)	850 (21)	1.21 (0.88–1.67)	97 (30)	465 (23)	1.26 (0.80–1.98)	55 (22)	385 (19)	1.14 (0.71–1.82)
Missing	10	73		4	34		6	39	
All	576	4,105		324	2,066		252	2,039	
Beer (servings)									
0–3/month	398 (73)	3,100 (79)	1.00*^d^*	189 (61)	1,333 (67)	1.00*^d^*	209 (90)	1,767 (91)	1.00*^d^*
1–6/week	54 (10)	336 (8)	1.28 (0.94–1.76)	42 (14)	239 (12)	1.25 (0.87–1.81)	12 (5)	97 (5)	1.31 (0.70–2.48)
≥1 per day	91 (17)	508 (13)	1.41 (1.09–1.84)	77 (25)	429 (21)	1.28 (0.95–1.72)	14 (6)	79 (4)	1.67 (0.92–3.05)
Missing	33	161		16	65		17	96	
All	576	4,105		324	2,066		252	2,039	
Wine (glasses)									
0–3/month	444 (81)	3,290 (83)	1.00*^d^*	241 (79)	1,631 (82)	1.00*^d^*	203 (85)	1,659 (84)	1.00*^d^*
1–6/week	48 (9)	307 (8)	1.21 (0.87–1.67)	26 (8)	144 (7)	1.16 (0.75–1.82)	22 (9)	163 (8)	1.27 (0.78–2.05)
≥1 per day	55 (10)	357 (9)	1.04 (0.76–1.41)	40 (13)	206 (10)	1.20 (0.83–1.74)	15 (6)	151 (8)	0.76 (0.44–1.34)
Missing	29	151		17	85		12	66	
All	576	4,105		324	2,066		252	2,039	
Liquor (ounces)									
0–3/month	433 (78)	3,248 (82)	1.00*^d^*	217 (70)	1,489 (74)	1.00*^d^*	214 (88)	1,759 (89)	1.00*^d^*
1–6/week	40 (7)	328 (8)	0.86 (0.61–1.23)	24 (8)	234 (12)	0.67 (0.43–1.06)	16 (7)	94 (5)	1.47 (0.84–2.57)
≥1 per day	83 (15)	408 (10)	1.34 (1.03–1.75)	69 (22)	284 (14)	1.60 (1.18–2.17)	14 (6)	124 (6)	0.85 (0.47–1.51)
Missing	22	121		14	59		8	62	
All	576	4,105		324	2,066		252	2,039	
Total fat intake (g/week)									
< 236	108 (21)	906 (24)	1.00*^d^*	55 (19)	368 (20)	1.00*^d^*	53 (24)	538 (29)	1.00*^d^*
236–323	116 (23)	925 (25)	1.06 (0.80–1.41)	52 (18)	426 (23)	0.81 (0.54–1.22)	64 (29)	499 (27)	1.39 (0.94–2.05)
324–420	129 (25)	899 (24)	1.21 (0.92–1.60)	81 (28)	457 (25)	1.24 (0.85–1.80)	48 (22)	442 (24)	1.12 (0.74–1.71)
> 420	160 (31)	998 (27)	1.34 (1.03–1.75)	105 (36)	598 (32)	1.22 (0.85–1.74)	55 (25)	400 (21)	1.44 (0.96–2.16)
Missing	63	377		31	217		32	160	
All	576	4,105		324	2,066		252	2,039	
Total energy intake (calories/week)									
< 1,370	121 (24)	1,039 (28)	1.00*^d^*	60 (20)	433 (23)	1.00*^d^*	61 (28)	606 (32)	1.00*^d^*
1,371–1,710	111 (22)	967 (26)	0.96 (0.73–1.27)	49 (17)	456 (25)	0.77 (0.51–1.15)	62 (29)	511 (27)	1.16 (0.80–1.69)
1,711–2,050	127 (25)	760 (20)	1.44 (1.10–1.88)	73 (25)	372 (20)	1.41 (0.97–2.05)	54 (25)	388 (21)	1.44 (0.97–2.13)
> 2,050	154 (30)	962 (26)	1.30 (1.01–1.69)	111 (38)	588 (32)	1.39 (0.98–1.95)	43 (20)	374 (20)	1.10 (0.72–1.67)
Missing	63	377		31	217		32	160	
All	576	4,105		324	2,066		252	2,039	
Tap water (glasses/week)									
< 1	90 (16)	687 (17)	1.00*^d^*	44 (14)	354 (18)	1.00*^d^*	46 (19)	333 (17)	1.00*^d^*
1–7	128 (23)	1,001 (25)	0.97 (0.73–1.30)	74 (24)	536 (27)	1.08 (0.72–1.61)	54 (22)	465 (23)	0.87 (0.57–1.33)
7–21	202 (36)	1,247 (31)	1.16 (0.89–1.52)	125 (40)	658 (33)	1.47 (1.01–2.13)	77 (31)	589 (29)	0.87 (0.58–1.29)
> 21	139 (25)	1,075 (27)	0.94 (0.70–1.25)	71 (23)	463 (23)	1.18 (0.78–1.77)	68 (28)	612 (31)	0.72 (0.48–1.08)
Missing	17	95		10	55		7	40	
All	576	4,105		324	2,066		252	2,039	
Education (total years)									
< 9	95 (17)	652 (16)	1.00*^d^*	66 (21)	393 (19)	1.00*^d^*	29 (12)	259 (13)	1.00*^d^*
9–12	281 (50)	1,770 (44)	1.23 (0.96–1.59)	145 (46)	842 (41)	1.04 (0.75–1.43)	136 (56)	928 (46)	1.57 (1.02–2.42)
> 12	187 (33)	1,624 (40)	0.99 (0.75–1.30)	107 (34)	797 (39)	0.84 (0.60–1.19)	80 (33)	827 (41)	1.27 (0.80–2.01)
Missing	13	59		6	34		7	25	
All	576	4,105		324	2,066		252	2,039	
Age at menarche (years)									
< 12							32 (17)	329 (17)	1.00*^d^*
12–15							137 (71)	1,271 (67)	1.06 (0.70–1.60)
> 15							24 (12)	285 (15)	0.75 (0.43–1.32)
Missing							59	154	
All							252	2,039	
Parity									
0							31 (11)	221 (11)	1.00*^d^*
1							21 (8)	161 (8)	0.96 (0.52–1.76)
2							60 (24)	498 (24)	0.93 (0.58–1.49)
3							55 (22)	410 (20)	0.91 (0.56–1.47)
≥4							85 (34)	744 (37)	0.63 (0.40–0.98)
Missing							0	5	
All							252	2,039	

a Number of cases (percentage of total excluding cases with missing values).

b Overall ORs adjusted for age, sex, and province.

c Sex-specific ORs adjusted for age and province.

d Referent group.

e Percent decrease of present weight compared with maximum lifetime weight.

**Table 2:** ORs for pancreatic cancer in relation to the concentration of CDBPs in drinking water (based on the EHD data set, 30-year ETW, full OCMI imputation, 3-year latency).

	Male and female*^a^*			Male*^a^*			Female*^b^*		
CDBP concentration (μg/L)	Cases	Controls	OR (95% CI)	Cases	Controls	OR (95% CI)	Cases	Controls	OR (95% CI)
THM									
< 10	139	951	1.00*^c^*	77	472	1.00*^c^*	52	450	1.00*^c^*
10–20	122	995	0.88 (0.67–1.17)	74	442	0.95 (0.65–1.40)	36	513	0.74 (0.46–1.20)
20–50	159	1,134	1.07 (0.83–1.39)	81	589	0.90 (0.63–1.29)	62	500	1.19 (0.79–1.81)
> 50	56	429	0.86 (0.58–1.28)	40	221	0.93 (0.56–1.55)	14	197	0.53 (0.25–1.12)
All	476	3,509		272	1,724		164	1,660	
	*p*_trend_ = 0.61		*p*_hetero_ = 0.46	*p*_trend_ = 0.74		*p*_hetero_ = 0.95	*p*_trend_ = 0.20		*p*_hetero_ = 0.057
BDCM									
< 1	135	982	1.00*^c^*	72	493	1.00*^c^*	55	459	1.00*^c^*
1–3	128	968	1.00 (0.77–1.32)	63	498	0.86 (0.59–1.26)	53	432	1.15 (0.75–1.77)
3–5	114	852	0.95 (0.71–1.29)	68	396	0.96 (0.64–1.44)	32	427	0.81 (0.48–1.36)
> 5	99	707	0.91 (0.67–1.25)	69	337	1.06 (0.70–1.60)	24	342	0.65 (0.36–1.16)
All	476	3,509		272	1,724		164	1,660	
	*p*_trend_ = 0.54		*p*_hetero_ = 0.94	*p*_trend_ = 0.70		*p*_hetero_ = 0.80	*p*_trend_ = 0.095		*p*_hetero_ = 0.24
TCM									
< 3	114	686	1.00*^c^*	63	356	1.00*^c^*	42	311	1.00*^c^*
3–10	119	948	0.79 (0.59–1.06)	75	410	0.90 (0.60–1.36)	34	501	0.62 (0.37–1.03)
10–30	146	1,262	0.79 (0.60–1.05)	72	644	0.64 (0.43–0.95)	59	568	0.85 (0.54–1.36)
> 30	97	613	1.08 (0.78–1.50)	62	314	1.09 (0.70–1.70)	29	279	0.82 (0.46–1.46)
All	476	3,509		272	1,724		164	1,660	
	*p*_trend_ = 0.42		*p*_hetero_ = 0.13	*p*_trend_ = 0.86		*p*_hetero_ = 0.066	*p*_trend_ = 0.94		*p*_hetero_ = 0.32

*p*_trend_ is the *p*-value testing for linear trend in the exposure ORs based on the median THM level in each exposure group, and *p*_hetero_ is the *p*-value testing the hypothesis that all ORs are equal to 1.0.

a Full model adjusted for sex, age, province of recruitment, BMI, percent weight change, smoking, coffee, beer, liquor, total fat intake, and total energy intake.

b Full model for females also adjusted for age at first menstruation and number of pregnancies; 44 female cases and 128 female controls were excluded because of missing data on menstrual or pregnancy history.

c Referent group.

**Table 3:** ORs for pancreatic cancer in relation to THM concentrations in drinking water (based on the PMW data set, 30-year ETW, full OCMI imputation, varying latencies, mean THM exposure levels).

	Male and female*^a^*			Male*^a^*			Female*^b^*		
THM concentration (μg/L )	Cases	Controls	OR (95% CI)	Cases	Controls	OR (95% CI)	Cases	Controls	OR (95% CI)
3-Year latency									
< 10	117	880	1.00*^c^*	63	433	1.00*^c^*	46	422	1.00*^c^*
10–20	131	997	1.07 (0.80–1.43)	76	433	1.14 (0.76–1.70)	39	523	0.91 (0.56–1.48)
20–50	190	1,352	1.17 (0.91–1.52)	106	720	1.07 (0.75–1.52)	64	582	1.11 (0.72–1.71)
> 50	48	367	0.90 (0.58–1.40)	34	193	0.92 (0.53–1.62)	14	162	0.82 (0.37–1.80)
All	486	3,596		279	1,779		163	1,689	
	*p*_trend_ = 0.74		*p*_hetero_ = 0.50	*p*_trend_ = 0.72		*p*_hetero_ = 0.88	*p*_trend_ = 0.74		*p*_hetero_ = 0.77
8-Year latency									
< 10	111	803	1.00*^c^*	64	399	1.00*^c^*	40	380	1.00*^c^*
10–20	127	1,008	1.01 (0.75–1.35)	68	436	0.92 (0.61–1.38)	43	539	1.02 (0.62–1.66)
20–50	201	1,420	1.19 (0.91–1.54)	112	754	1.00 (0.70–1.43)	68	607	1.25 (0.80–1.95)
> 50	47	365	0.80 (0.51–1.24)	35	190	0.88 (0.51–1.51)	12	163	0.55 (0.23–1.28)
All	486	3,596		279	1,779		163	1,689	
	*p*_trend_ = 0.45		*p*_hetero_ = 0.21	*p*_trend_ = 0.69		*p*_hetero_ = 0.92	*p*_trend_ = 0.28		*p*_hetero_ = 0.22
13-Year latency									
< 10	110	746	1.00*^c^*	60	372	1.00*^c^*	41	351	1.00*^c^*
10–20	123	1,012	0.88 (0.67–1.18)	72	435	0.97 (0.65–1.46)	41	542	0.82 (0.50–1.33)
20–50	205	1,479	1.11 (0.85–1.44)	110	782	0.98 (0.68–1.41)	70	639	1.12 (0.72–1.74)
> 50	48	359	0.77 (0.49–1.19)	37	190	0.91 (0.52–1.56)	11	157	0.46 (0.19–1.10)
All	486	3,596		279	1,779		163	1,689	
	*p*_trend_ = 0.39		*p*_hetero_ = 0.17	*p*_trend_ = 0.72		*p*_hetero_ = 0.99	*p*_trend_ = 0.16		*p*_hetero_ = 0.13

*p*_trend_ is the *p*-value testing for linear trend in the exposure ORs based on the median THM level in each exposure group, and *p*_hetero_ is the *p*-value testing the hypothesis that all ORs are equal to 1.0.

a Full model adjusted for sex, age, province of recruitment, BMI, percent weight change, smoking, coffee, beer, liquor, total fat intake, and total energy intake.

b Full model for females also adjusted for age at first menstruation and number of pregnancies; 44 female cases and 128 female controls were excluded because of missing data on menstrual or pregnancy history.

c Referent group.

**Table 4:** Effect of THM exposure on risk of pancreatic cancer (based on the PMW data set, full OCMI imputation, weighted by drinking water intake, mean THM exposure levels).

	Male and female*^a^*			Male*^a^*			Female*^b^*		
THM concentration (μg/L)	Cases	Controls	OR (95% CI)	Cases	Controls	OR (95% CI)	Cases	Controls	OR (95% CI)
Quartile 1*^c^*	115	885	1.00*^d^*	64	442	1.00*^d^*	42	412	1.00*^d^*
Quartile 2	116	915	1.07 (0.80–1.43)	64	459	0.94 (0.63–1.40)	38	425	1.09 (0.67–1.78)
Quartile 3	125	909	1.15 (0.86–1.53)	65	425	1.01 (0.68–1.51)	47	454	1.20 (0.75–1.92)
Quartile 4A	70	447	1.18 (0.85–1.65)	46	228	1.23 (0.80–1.91)	19	198	0.95 (0.52–1.74)
Quartile 4B	60	440	0.90 (0.62–1.33)	40	225	0.87 (0.53–1.43)	17	200	0.77 (0.38–1.55)
All	486	3,596		279	1,779		163	1,689	
	*P*_trend_ = 0.72		*P*_hetero_ = 0.64	*P*_trend_ = 0.79		*P*_hetero_ = 0.69	*P*_trend_ = 0.46		*P*_hetero_ = 0.77

*p*_trend_ is the *p*-value testing for linear trend in the exposure ORs based on the median THM level in each exposure group, and *p*_hetero_ is the *p*-value testing the hypothesis that all ORs are equal to 1.0.

a Full model adjusted for sex, age, province of recruitment, BMI, percent weight change, smoking, coffee, beer, liquor, total fat intake, and total energy intake.

b Full model for females also adjusted for age at first menstruation and number of pregnancies; 44 female cases and 128 female controls were excluded because of missing data on menstrual or pregnancy history.

c Quartiles were defined based on distribution of exposure estimates in combined sample based on the 30-year ETW with 3-year latency weighted by self-reported intake of drinking water; quartile 4 was subdivided into two groups based on the median exposure in the quartile.

d Referent group.
